# Insights into the Dual Activity of SIVmac239 Vif against Human and African Green Monkey APOBEC3G

**DOI:** 10.1371/journal.pone.0048850

**Published:** 2012-11-26

**Authors:** Ritu Gaur, Klaus Strebel

**Affiliations:** 1 Faculty of Life Sciences and Biotechnology, South Asian University, Akbar Bhawan, Chanakyapuri, New Delhi, India; 2 Laboratory of Molecular Microbiology, Viral Biochemistry Section, National Institute of Allergy and Infectious Diseases, National Institutes of Health, Bethesda, Maryland, United States of America; Harvard Medical School, United States of America

## Abstract

Human immunodeficiency virus type 1 (HIV-1) Vif is essential for viral evasion of the host antiviral protein APOBEC3G (APO3G). The Vif protein from a distantly related African green monkey (Agm) simian immunodeficiency virus (SIVagm) is unable to suppress the antiviral activity of human APO3G but is active against Agm APO3G. SIVmac Vif on the other hand, possesses antiviral activity against both human and Agm APO3G. In this study, we were interested in mapping domains in SIVmac Vif that are responsible for its dual activity against human and Agm APO3G. We constructed a series of Vif chimeras by swapping domains in SIVmac Vif with equivalent regions from SIVagm Vif and determined their activity against human and Agm APO3G. We found that replacing any region in SIVmac Vif by corresponding fragments from SIVagm Vif only moderately reduced the activity of the chimeras against Agm APO3G but in all cases resulted in a severe loss of activity against human APO3G. These results suggest that the domains in SIVmac Vif required for targeting human and Agm APO3G are distinct and cannot be defined as linear amino acid motifs but rather appear to depend on the overall structure of full-length SIVmac Vif.

## Introduction

The human immunodeficiency virus type 1 (HIV-1) Vif protein plays a crucial role during the viral life cycle by regulating virion infectivity and *in vivo* pathogenesis. Vif counteracts a cellular factor identified as APOBEC3G (APO3G) [1]. APO3G is a member of the APOBEC superfamily, which share a cytidine deaminase motif [2], [3]. In the absence of Vif, APO3G is incorporated into virus particles, where it causes editing of the viral cDNA during reverse transcription [4]–[7]. The conversion of deoxycytidine to deoxyuridine on the minus-strand cDNA results in deoxyguanine-to-deoxyadenine changes on the viral plus-strand cDNA to yield highly mutated viral genomes. Virus replication may be inhibited through accumulation of mutations in the viral genome or through degradation of the deaminated viral cDNA via a cellular DNA repair mechanism [8]. Alternatively, APO3G may inhibit virus replication through deamination-independent mechanisms [9]–[17]. The Vif protein reduces cellular expression of APO3G and its incorporation into virions. The precise mechanism through which Vif accomplishes this task is still under investigation. However, there is strong evidence that there is a physical interaction between Vif and APO3G, which can lead to APO3G degradation by the host proteasome machinery [2]. Recent reports have suggested that Vif recruits a transcription factor CBF-ß to degrade APO3G [18]–[20]. Other studies, however, suggest that intracellular degradation may not be the sole mechanism by which Vif neutralizes APO3G's antiviral activity [6], [18]–[21].

Mutational analysis of Vif has led to the characterization of several distinct binding domains in Vif for assembly of an E3 ubiquitin ligase complex, as well as for interaction with APO3G. One of the domains involved is a highly conserved motif near the C terminus of Vif, referred to as the SLQ motif. The exact APO3G binding domain in Vif is still incompletely defined and most likely consists of several discontinuous subdomains [22]–[29]. Mutations in HIV-1 Vif at positions 14–17 allowed HIV-1 Vif to counteract hAPO3G and rhAPO3G [30]. Site-directed mutagenesis identified residues 40 to 44 (YRHHY) in HIV-1 Vif as important for binding of APO3G [27]–[32]. Moreover, K26 in Vif was found to be critical for APO3G interaction [33]–[35]. In addition, a stretch of hydrophobic amino acids comprising residues 69 to 72 in HIV-1 Vif is important for interaction with APO3G [36], [37]. Finally, analysis of patient-derived HIV-1 Vif sequences demonstrated the importance of residues K22, Y40, and E45 for APO3G recognition [38].

Aside from HIV, APO3G was found to target a broad range of retroviruses as well as retroid viruses and retrotransposons. These include simian immunodeficiency virus (SIV), equine infectious anemia virus (EIAV), murine leukemia virus (MLV), Hepatitis B virus (HBV), the Ty1 retrotransposon, and intracisternal A-particles [5], [39]–[44]. Vif defective HIV-1 virus is blocked by APO3G from human, rhesus macaque, African Green Monkey (Agm), and mouse [6]. In contrast, the ability of Vif to block the antiviral activity of APO3G is species-specific and several independent studies mapped a determinant of this species specificity to amino acid 128 of APO3G [45]–[48]. Indeed, mutation of amino acid 128 (D128K) in human APO3G rendered the protein insensitive to HIV-1 Vif but made it sensitive to SIVagm Vif. The insensitivity of the APO3G D128K mutant to HIV-1 Vif may be due to lack of interaction of HIV-1 Vif with APO3G as several groups noted a severe effect of such mutation on the co-immunoprecipitation of Vif and APO3G [45]–[47] . Recently, a single amino acid in human APO3F has also been shown to alter susceptibility to HIV-1 Vif [49].

HIV-1 and SIVagm Vif are mono-specific and can only inactivate APO3G of the species from which they are derived. In contrast, SIVmac Vif can inhibit both human and Agm APO3G [6]. In this study we were interested in elucidating the mechanism by which SIVmac Vif exerts its dual activity against human and Agm APO3G. Thus, in contrast to a previous study, which aimed at extending the host range of HIV-1 Vif [30], the scope of the current study was to expand the host range of the mono-specific SIVagm Vif. To map determinants in SIVmac Vif required for its activity against human and Agm APO3G, we constructed a series of SIVmac/agm Vif chimeras and tested their ability to target human and Agm APO3G. Our results demonstrate that the ability of SIVmac Vif to target human APO3G is highly sensitive to the exchange of regions in Vif by fragments derived from SIVagm Vif. In contrast, inhibition of Agm APO3G by SIVmac Vif is relatively insensitive to domain swapping. These results suggest that the determinants for functional interaction of SIVmac Vif with human and Agm APO3G are distinct and cannot be defined as linear amino acid motifs but rather appear to depend on the overall structure of full-length SIVmac Vif and may involve multiple contact points.

## Methods

### Ethics Statement

This study was carried out in strict accordance with the recommendations in the Guide for the Care and Use of Laboratory Animals of the National Institutes of Health. The protocol (LMM32) was approved by National Institute of Allergy and Infectious Diseases, Division of Intramural Research Animal Use and Care Committee (PHS Assurance #A4149-01). Animals were housed and procedures were carried out at the NIH facilities in the US in accordance with all local, state and Federal laws governing in vivo research, in facilities fully accredited by the Association and Accreditation of Laboratory Animal Care International (AAALAC). Details of animal welfare and steps taken to ameliorate suffering are included in the methods section of the manuscript.

### Plasmids

For transient expression of HIV-1 Vif, subgenomic expression vectors based on pNL-A1 [50] were employed. This plasmid expresses HXB2 Vif and all HIV-1 proteins except for *gag* and *pol* products. For the expression of NL-43 Vif, SIVmac239 Vif, and SIVagm9063 (Genbank L40990) Vif, the *vif* genes of these isolates were amplified by PCR from the respective full-length molecular clones and subcloned into the BssHII/EcoRI sites of pNL-A1 resulting in pNL-A1/43Vif, pNL-A1/macVif, and pNL-A1/agmVif, respectively. Because of this cloning strategy, the *vpr* gene was inactivated in these constructs. All other HIV-1 proteins (i.e. Tat, Rev, Vpu, Env, and Nef) are expressed as in pNL-A1. A *vif*-defective variant of pNL-A1, pNL-A1vif (-), was constructed by deleting a *Nde*I/*Pfl*MI fragment in *vif*, resulting in a translational frame-shift following amino acid 28 [51]. For virus production, a *vif*-defective variant of the SIVmac carrying a deletion in *vif* was constructed by ligation of two SphI-digested fragments derived from p7–21 and p239SpE3′ obtained from Ronald Desrosiers through the NIH AIDS Research & Reference Reagent Program. The resulting construct, pSIVmac239Vif(-), expresses all viral proteins except Vif. Plasmid pcDNA-hApo3G and pcDNA-Agm-Apo3G are vectors for the expression of human APO3G and Agm APO3G, respectively, under the control of the CMV immediate early promoter. These proteins carry a C-terminal Myc-His epitope tag. Construction of pcDNA-hApo3G was described previously [22]. Plasmid pcDNA-Agm-Apo3G was derived from pc-AGM-Apo3G-HA (gift of Nathaniel Landau) by PCR amplification of the Agm APO3G gene and subcloning the PCR product into pcDNA3.1(-)MycHis (Invitrogen Corp., Carlsbad CA).

### Construction of Vif chimeras

SIVmac chimeras were generated by PCR-based mutagenesis using SIVmac239, SIVagm9063, and NL4-3 Vif expression plasmids as templates. Briefly, 5′ and 3′ fragments were amplified separately using primers specific for the overlapping region in combination with a 5′ external primer carrying a *Bss*HII site (for the 5′ fragment) or a 3′ external primer carrying an *Eco*RI site (for the 3′ fragment). The 5′ and 3′ primary PCR products were purified by gel electrophoresis and mixed at equimolar ratios. A second-round PCR reaction was then performed using 5′ and 3′ external primers from the first PCR reaction and the primary PCR products as template. The resulting full-length PCR products were cleaved with *Eco*RI and *Bss*HII restriction enzymes and cloned into pNL-A1 cleaved with the same enzymes. All constructs were confirmed by DNA sequence analysis. The expression of all chimeric proteins was verified by immunoblotting using antibodies to HIV-1, SIVmac239, or SIVagm9063 Vif proteins.

### Antisera

Exogenously expressed APO3G was identified using a polyclonal rabbit peptide serum (APOC17). The serum reacts with human and Agm APO3G and is available through the NIH AIDS Research & Reference Reagent Program (Cat No: 10082). Polyclonal antibodies against the Vif proteins of SIVagm9063 and SIVmac239 were prepared by immunizing rabbits with purified recombinant proteins.

### Virus and Rhesus Macaques

Serum from a rhesus monkey infected with SIVmac239 was used for the identification of SIVmac239 capsid protein and was a generous gift of Dr. Malcolm Martin (LMM, NIAID, NIH, Bethesda) [52]. The origin and preparation of the tissue culture-derived SIVmac239 stocks from molecular clones have been described previously [53], [54]. Rhesus macaques (Macaca mulatta) were maintained in accordance with the guidelines of the Committee on Care and Use of Laboratory Animals [55] and were housed in a biosafety level 2 facility; biosafety level 3 practices were followed. All studies were approved by NIAID Institutional Animal Care and Use Committees. Four healthy Rhesus Macaques (Macaca mulatta) were injected with the SIVmac239 clone intravenously at 1000 times the 50% tissue culture infectious dose (1000 TCID50). Peripheral blood samples were obtained from the animals on day 7 and day 10 post infection.

All procedures were performed under appropriate anesthesia to alleviate pain and minimize suffering as published by the National Institutes of Health Animal Resource Advisory Committee (ARAC).

### Tissue culture and transfections

HeLa cells were propagated in Dulbecco's modified Eagles medium (DMEM) containing 10% fetal bovine serum (FBS). For transfection of HeLa cells, cells were grown in 25 cm^2^ flasks to about 80% confluency. Cells were transfected using LipofectAMINE PLUS™ (Invitrogen Corp, Carlsbad CA) following the manufacturer's recommendations. Cell were transfected with 3 µg of the molecular clone DNA. A total of 5–6 µg of plasmid DNA per 25 cm^2^ flask was used. Cells were harvested 48 hr post-transfection. LuSIV cells were maintained in complete RPMI 1640 medium supplemented with 10% FBS and hygromycin B (300 µg/ml). These cells were obtained from Janice Clements through the NIH AIDS Research & Reference Reagent Program (Cat. No. 5460) [24].

Virus stocks were prepared by transfection of HeLa cells with appropriate plasmid DNAs. Virus-containing supernatants were harvested 24 h after transfection. Cellular debris was removed by centrifugation (3 min, 1500 rpm) and clarified supernatants were filtered (0.45 µM) to remove residual cellular contaminations. Filtered virus stocks were further purified and concentrated by pelleting through 20% sucrose (75 min, 4°C at 35,000 rpm in an SW41 rotor) [22].

### Immunoblotting

For immunoblot analysis of intracellular proteins, whole cell lysates were prepared as follows: Cells were washed once with PBS, suspended in PBS (400 µl/10^7^ cells), and mixed with an equal volume of sample buffer (4% sodium dodecyl sulfate, 125 mM Tris-HCl, pH 6.8, 10% 2-mercaptoethanol, 10% glycerol, and 0.002% bromophenol blue). Proteins were solubilized by boiling for 10 to 15 min at 95°C with occasional vortexing of the samples to shear chromosomal DNA. Residual insoluble material (generally non-existent) was removed by centrifugation (2 min, 16,000× g in an Eppendorf Minifuge). Cell lysates were subjected to SDS-PAGE; proteins were transferred to PVDF membranes and reacted with appropriate antibodies as described in the text. Membranes were then incubated with horseradish peroxidase-conjugated secondary antibodies (Amersham Biosciences, Piscataway NJ) and visualized by enhanced chemiluminescence (ECL, Amersham Biosciences).

### Co-immunoprecipitation of Vif and APO3G

HeLa cells were transfected with 1 µg each of pcDNA-hApo3g Myc/His and 4 µg of the various Vif expression vectors along with 0.5 µg of Cul5ΔRbx plasmid. Cells were lysed 24 hr post transfection in 300 µl of 0.5% Triton X-100 buffer (50 µM Tris, pH 7.8, 150 mM NaCl, 0.5% Triton X-100). Immunoprecipitation was carried out using a polyclonal antibody to a C-terminal Myc epitope tag in APO3G. The antibody was adsorbed on Protein A-Sepharose beads (Sigma-Aldrich, St. Louis MO) for 1 hr at 4°C. Beads were then washed once with Triton-Wash Buffer (50 mM Tris 7.4, 300 mM NaCl, 0.1% Triton X100) and 200 µl of cell lysate was added to the beads and incubated for 1 hr at 4°C. The beads were then washed 3 times with Triton-Wash Buffer. Bound proteins were solubilized by boiling the samples in 100 µl of sample buffer (4% sodium dodecyl sulfate, 125 mM Tris-HCl, pH 6.8, 10% 2-mercaptoethanol, 10% glycerol, and 0.002% bromophenol blue) and separated on 12.5% polyacrylamide-SDS gels. The proteins were transferred to PVDF membranes and reacted with a cocktail of SIVagm Vif and SIVmac Vif-specific antibodies. Membranes were then incubated with horseradish peroxidase-conjugated secondary antibodies and visualized by enhanced chemiluminescence (ECL).

### Infectivity assay

To determine viral infectivity, virus stocks were normalized for equal reverse transcriptase activity and used to infect LuSIV cells (5×10^5^) in a 24-well plate in a total volume of 1.2 to 1.4 ml. LuSIV cells are derived from CEMx174 cells and contain a luciferase indicator gene under the control of the SIVmac239 LTR [56]. These cells were obtained through the NIH AIDS Research and Reference Reagent Program and were maintained in complete RPMI 1640 medium supplemented with 10% FBS and hygromycin B (300 µg/ml). Cells were infected for 24 hr at 37°C. Cells were then harvested and lysed in 150 µl of Promega 1× reporter lysis buffer (Promega Corp., Madison WI). To determine the luciferase activity in the lysates, 50 µl of each lysate was combined with luciferase substrate (Promega Corp., Madison WI) by automatic injection and light emission was measured for 10 seconds at room temperature in a luminometer (Optocomp II, MGM Instruments, Hamden CT).

## Results

### Inhibition of human and Agm APO3G by HIV-1 Vif, SIVmac Vif, and SIVagm Vif

**Figure 1:**
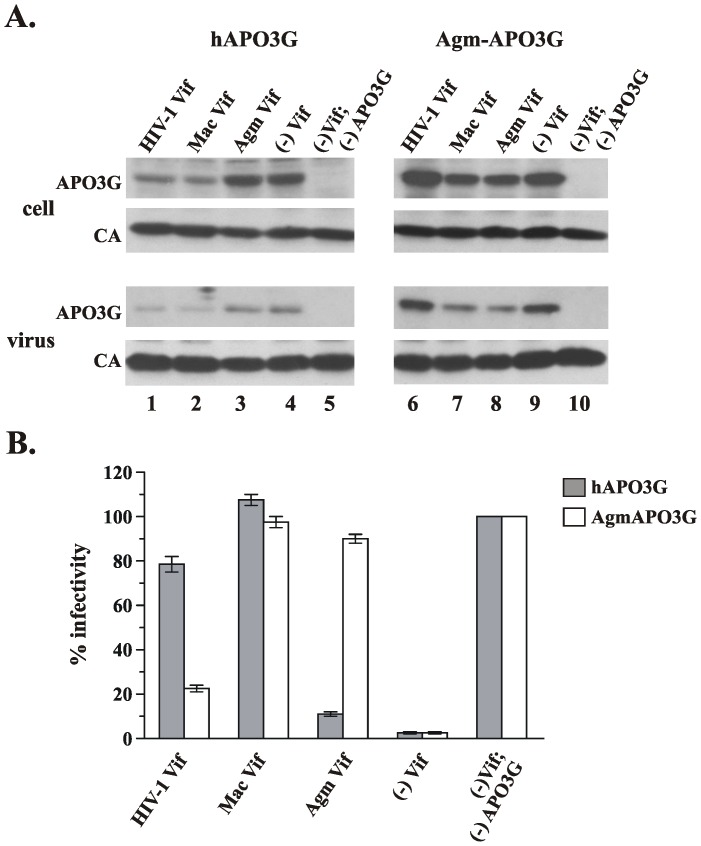
Activity of HIV-1, SIVagm and SIVmac Vif against human and Agm APO3G. (**A**) HeLa cells were transfected with the Vif-defective proviral clone SIVmac Vif(-) and pCDNA-hAPO3G (left panel) or pCDNA-Agm-Apo3G vector (right panel). In addition, vectors encoding HIV-1 Vif (pNL-A1/43Vif, lanes 1 & 6), Mac Vif (pNL-A1macVif, lanes 2 & 7), or Agm Vif (pNL-A1agmVif, lanes 3 & 8) were co-transfected. Vif-negative samples (lanes 4 & 9) and APO3G-negative samples (lanes 5 & 10) were included as controls. Total amount of DNA was adjusted to 5.5 µg/5×10^6^ cells using pNL-A1vif(-) vector DNA. Whole-cell lysates and concentrated viral extracts were prepared 48 hr after transfection and aliquots from each sample were analyzed by immunoblotting for the presence of APO3G (top panel) or capsid protein (CA, lower panel). (**B**) Cell-free filtered supernatants from the cultures shown in panel A were normalized for reverse transcriptase activity and used for the infection of LuSIV indicator cells. Infected cells were harvested 24 hr later and analyzed for the expression of Tat-induced luciferase activity. Results are expressed relative to the virus prepared in the absence of APO3G (defined as 100%). Bars reflect the median and error bars indicate the range calculated from two independent infections.

It has previously been reported that HIV-1 Vif inhibits the activity of human APO3G but not Agm APO3G, whereas SIVagm Vif is active against Agm APO3G but not human APO3G [6], [45]–[47], [57], [58]. SIVmac Vif, on the other hand, was found to inhibit both human and Agm APO3G [47], [48]. To confirm these results, we compared the activity of HIV-1 Vif, SIVmac Vif, and SIVagm Vif on human and Agm APO3G in the backbone of a *vif*-defective SIVmac239 virus. HeLa cells were transfected with pcDNA-hApo3G or pcDNA-Agm-Apo3G and either pNL-A1/43Vif, pNL-A1/macVif, or pNL-A1/agmVif vector DNA in addition to DNA encoding *vif*-defective SIVmac239. HIV-1 Vif reduced cellular expression and packaging of human APO3G but not Agm APO3G (Fig. 1A, lanes 1 & 6) when compared to Vif-negative samples (Fig. 1A, lanes 4 & 9). SIVagm Vif, on the other hand was active only against Agm APO3G (Fig. 1A, lanes 3 & 8). In contrast, SIVmac Vif was able to reduce intracellular expression and inhibit the packaging of both human and Agm APO3G (Fig. 1A, lanes 2 & 7).

The ability of HIV-1, SIVmac, and SIVagm Vif to rescue the infectivity of SIVmac239 produced in the presence of human or Agm APO3G was tested in a single cycle assay as described in Materials and Methods. The infectivity data correlated well with the cellular activity of Vif on APO3G steady-state levels. As expected, HIV-1 Vif and Agm Vif were largely mono-specific and could only inhibit the antiviral activity of human and Agm APO3G, respectively (Fig. 1B). A partial rescue of viral infectivity was observed for HIV-1 Vif in the presence of Agm APO3G (Fig. 1B, open bar). In contrast, SIVmac Vif was active against both human and Agm APO3G and directed the production of fully infectious SIVmac in the presence of either deaminase. Experiments performed in the backbone of vif-defective HIV-1 viruses yielded very similar results (data not shown). Thus, the mono-specific function of HIV-1 Vif and SIVagm Vif and the dual activity of SIVmac Vif against human and Agm APO3G is a general property of these Vif proteins and not restricted by the viral background.

### Construction and expression of SIVmac Vif chimeras

To identify residues in SIVmac Vif responsible for its activity against human and/or Agm APO3G, we constructed a panel of SIVmac/agm chimeras. Our aim was to identify the smallest segment of SIVmac Vif that when substituted by SIVagm Vif would result in a loss of activity against human APO3G while retaining activity against Agm APO3G. We expected the SIVmac/agm chimeras to retain activity against Agm APO3G since SIVmac Vif and SIVagm Vif are both active against Agm APO3G.

**Figure 2:**
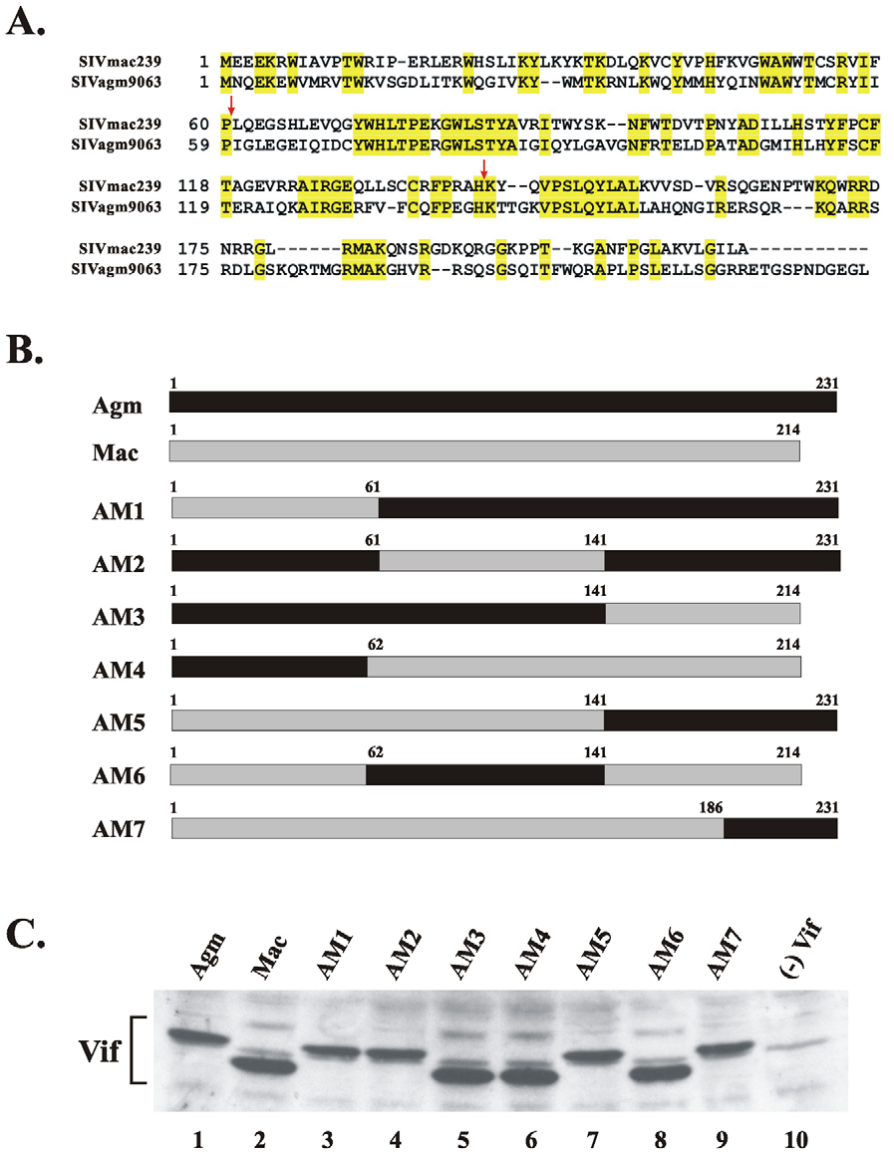
Construction and expression of SIVmac/agm Vif chimeras. (**A**) Alignment of SIV Mac239 and SIV Agm9063 Vif. The fusion points between chimeras are indicated as red arrows. (**B**) SIVmac/agm chimeras were constructed by PCR based mutagenesis as described in the Methods section. These mutants are labeled as AM1–AM7. The numbers above each construct indicate the amino acid positions for each fusion point. (**C**) HeLa cells were transfected with 5 µg of plasmid DNA encoding wild type SIVagm9063 Vif (lane 1), wild type SIVmac239 Vif (lane 2) or the various chimeras (AM1–AM7, lanes 3–9). A Vif-negative sample was included as a control (lane 10). Whole-cell lysates were prepared 48 hr after transfection and aliquots from each sample were analyzed by immunoblotting using an antibody cocktail against SIVmac239 and SIVagm9063 Vif proteins.

The borders between individual segments (residues 61/62 and 141) were chosen to reside in regions of local homology between SIVagm and SIVmac Vif (Fig. 2A). Also, care was taken not to interrupt known functional motifs in Vif such as the proteolytic processing site [59] or the Cul5 binding motif [60]. A schematic representation of the chimeras is shown in figure 2B. The expression of all the chimeras was verified by SDS-PAGE followed by immunoblot analysis with an antibody cocktail recognizing both SIVmac and SIVagm Vif (Fig. 2C). Because of differences in their C-terminal regions, the Vif proteins of SIVagm and SIVmac differ in their sizes (231 and 214 residues, respectively). As a result, the proteins migrate with different mobilities in the gel (Fig. 2C, lanes 1 & 2). Accordingly, chimeras AM1, AM2, AM5, and AM7, containing the C-terminal domain of SIVagm migrated similar to SIVagm (Fig. 2C, lanes 3, 4, 7, 9) while chimeras AM3, AM4, and AM6, containing the C-terminus of SIVmac migrated with similar mobility than the parental SIVmac protein (Fig. 2C, lanes 5, 6, 8).

### SIVmac/agm Vif chimeras fail to inhibit human APO3G

**Figure 3:**
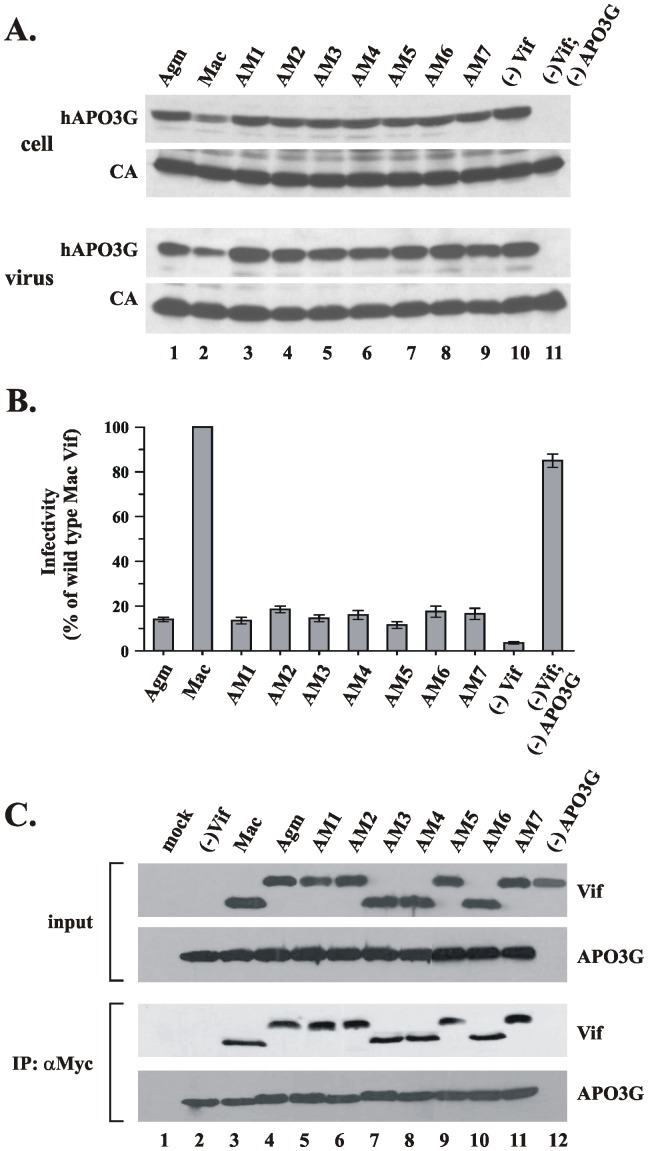
SIVmac/agm Vif chimeras are inactive against human APO3G. (**A**) HeLa cells were transfected to express constant amounts of Vif-defective SIVmac Vif(-) and human APO3G along with SIVagm Vif (lane 1), SIVmac Vif (lane 2), or the SIVmac/agm chimeras AM1–AM7 (lanes 3–9). Vif-negative (lane 10) or APO3G-negative samples (lane 11) were included as controls. Total amount of DNA in all samples was adjusted to 5.5 µg/5×10^6^ cells using pNL-A1vif(-) vector. Whole-cell lysates and concentrated viral extracts were prepared 48 hr after transfection and aliquots from each sample were analyzed by immunoblotting for the presence of APO3G (top panel) or Gag products (lower panel). (**B**) Cell-free filtered supernatants from the cultures shown in panel A were normalized for reverse transcriptase activity and used for the infection of LuSIV indicator cells. Infected cells were harvested 24 hr later and analyzed for the expression of Tat-induced luciferase activity. Results are expressed relative to the virus prepared in the presence of wild type SIVmac239 Vif (Mac Vif), which was defined as 100%. Bars reflect the median and error bars indicate the range calculated from two independent infections. (**C**). HeLa cells were transfected with pcDNA-hAPO3G and vectors encoding wild type SIVmac Vif, SIV agm Vif, or SIVmac/agm Vif chimeras in the presence of Cul5ΔRbx. Cell lysates were prepared 48 hr after transfection and subjected to immunoprecipitation with a Myc-specific rabbit polyclonal antibody as detailed in Methods. Total lysates (input control) and immunoprecipitated samples were then analyzed by immunoblotting with either Vif or APO3G -specific antibodies.

We next checked the activity of SIVmac/agm Vif chimeras against human APO3G. Hela cells were transfected with *vif*-defective SIVmac proviral DNA along with pcDNA-hApo3G and either wild type SIVagm or SIVmac Vif or one of the chimeric constructs described in figure 2. Protein expression, virus production, and packaging of APO3G into SIVmac virions was determined 48 hr post-transfection by immunoblotting of whole cell lysates and concentrated virus preparations using antibodies to APO3G (Fig. 3A, hAPO3G) or a SIVmac-specific serum (Fig. 3A, CA). As expected, SIVmac Vif caused a reduction of the cellular human APO3G levels and reduced the packaging of the enzyme into SIVmac particles (Fig. 3A, lane 2). In contrast, Agm Vif was unable to reduce expression of human APO3G and did not inhibit its packaging into SIVmac particles (Fig. 3A, lane 1).

The SIVmac/agm chimera AM4 in which the N-terminal domain of SIVmac Vif was replaced by that of SIVagm Vif was no longer active against human APO3G (Fig. 3A, lane 6). This indicated that the N-terminal domain of SIVmac Vif might be responsible for its activity against human APO3G. However, exchange of C-terminal (AM5; lane 7) and central domains of SIVmac Vif (AM6; lane 8) by SIVagm sequences resulted in a similar loss of activity against human APO3G. In fact, replacement of a 45 amino acid segment from the highly divergent C-terminus of SIVmac Vif by SIVagm sequences was sufficient to eliminate the activity against human APO3G (AM7; Fig. 3A, lane 9). Replacing both the N- and C-terminal region in SIVmac Vif by SIVagm sequences (AM2; Fig. 3A, lane 4) or exchanging larger N- and C-terminal portions in SIVmac Vif (AM3 and AM1, respectively; Fig. 3A, lanes 3 & 5) further resulted in a loss of activity against human APO3G. Thus, replacement of any segment in SIVmac Vif by corresponding sequences of SIVagm Vif invariably resulted in the loss of activity against human APO3G.

These findings are further supported by the results from a single cycle infectivity assay, in which the infectivity of the viruses shown in figure 3A was determined (Fig. 3B). Consistent with the results from figure 3A, wild type SIVmac Vif but not SIVagm Vif was able to produce infectious SIVmac viruses in the presence of human APO3G. In contrast, all of the SIVmac/agm Vif chimeras behaved like SIVagm Vif and were unable to effectively counteract the antiviral activity of human APO3G.

The inhibition of APO3G's antiviral activity requires a physical interaction with Vif [1], [17], [25], [36]. Yet, the region in Vif required for the interaction with APO3G remains ill defined. It is indeed possible that the lack of activity of the SIVmac/agm Vif chimera is caused by their inability to interact with human APO3G. This was tested by co-immunoprecipitation analysis. HeLa cells were transfected with pcDNA-hApo3G and vectors encoding wild type SIVmac Vif or SIVmac/agm Vif chimeras. Cell lysates were prepared 48 hr after transfection and subjected to immunoprecipitation using a rabbit polyclonal antibody to the C-terminal Myc epitope tag in human APO3G. Whole cell lysates and immunoprecipitated samples were then analyzed by immunoblotting using an antibody cocktail against SIVmac and SIVagm Vif (Fig. 3C). The results show that the SIVmac/agm chimeras interacted equally well with human APO3G than wild type SIVmac or SIV agm Vif. Of note, Agm Vif was not immunoprecipitated by the Myc antibodies in the absence of APO3G (Fig. 3C, lane 12), attesting to the specificity of the experiment. Thus, the inability of the Vif chimeras to target human APO3G cannot be explained by a possible loss of its ability to interact with human APO3G.

### SIVmac/agm Vif chimeras can target Agm APO3G

**Figure 4:**
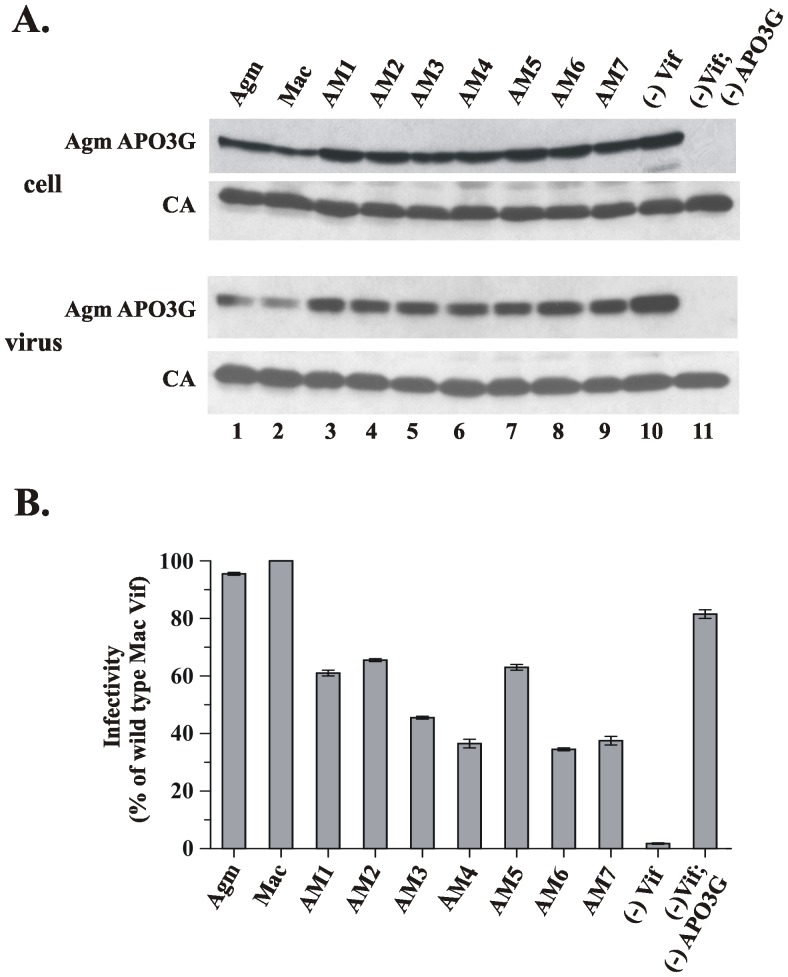
SIVmac/agm Vif chimeras retain activity towards Agm APO3G. (**A**) HeLa cells were transfected to express constant amounts of Vif-defective SIVmac Vif(-) and Agm APO3G along with SIVagm Vif (lane 1), SIVmac Vif (lane 2), or the SIVmac/agm chimeras AM1-AM7 (lanes 3–9). Vif-negative (lane 10) or APO3G-negative samples (lane 11) were included as controls. Total amount of DNA in all samples was adjusted to 5.5 µg/5×10^6^ cells using pNL-A1vif(-) vector. Whole-cell lysates (Cell) and concentrated viral extracts (Virus) were prepared 48 hr after transfection and aliquots from each sample were analyzed by immunoblotting for the presence of APO3G (Agm APO3G) or Gag products (CA). (**B**) Cell-free filtered supernatants from the cultures shown in panel A were normalized for reverse transcriptase activity and used for the infection of LuSIV indicator cells. Infected cells were harvested 24 hr later and analyzed for the expression of Tat-induced luciferase activity. Results are expressed relative to the virus prepared in the presence of wild type SIVmac239 Vif (defined as 100%). Bars reflect the median and error bars indicate the range calculated from two independent infections.

The inability of the SIVmac/agm chimeras to target human APO3G could reflect a general lack of activity or could be due to a selective loss of activity towards human APO3G. To explore that question, we next tested the activity of the SIVmac/agm chimeras against Agm APO3G. The experiment was performed analogous to figures 3A and 3B on the background of *vif*-defective SIVmac239 to analyze the effects of the Vif chimeras on Agm APO3G expression and encapsidation (Fig. 4A) or antiviral activity (Fig. 4B). As expected, wild type SIVagm Vif and SIVmac Vif reduced the cellular expression of Agm APO3G and reduced the packaging of Agm APO3G into SIVmac virions (Fig. 4A, lanes 1 & 2). The chimeric Vif molecules induced a moderate reduction of the cellular expression and packaging of Agm APO3G when compared to the Vif-negative control (Fig. 4A; Agm APO3G, compare lanes 3–9 with lane 10). Interestingly, all of the chimeras were able to rescue at least in part the infectivity of SIVmac virions (Fig. 4B). In particular, chimeras AM1, AM2, and AM5 retained about 60–65% of wild type activity. These chimeras contain the C-terminal domain of SIVagm (residues 141 to 231; see Fig. 2A). Chimeras containing the SIVmac C-terminus (AM3, AM4, and AM6) or a chimera containing a portion of the SIVagm C-terminal domain (AM7) were slightly more restricted in their activity against Agm APO3G but still retained up to 40% of wild type activity (Fig. 4B). These results suggest that exchanging domains in SIVmac Vif with corresponding domains of SIVagm Vif did not have the same debilitating effects observed with respect to human APO3G and only moderately affected the ability of the Vif chimeras to target Agm APO3G.

## Discussion

Lentiviral Vif proteins function in a host-specific manner [6], [58]. Indeed, recent studies demonstrated that human APO3G was inhibited by Vif from HIV-1 isolates but not by SIVagm Vif, whereas Agm APO3G was inhibited by Vif from SIVagm but only partially by HIV-1 Vif. Several independent reports suggested that a single amino acid at position 128 in APO3G (D128K) was responsible for the species-specific sensitivity of APO3G to Vif [45]–[48]. Most of these studies found that mutation of residue 128 in human APO3G prevented the binding to HIV-1 Vif [45]–[48] suggesting that this residue in APO3G constitutes part of a Vif binding motif. Accordingly, changing the lysine 218 residue in Agm APO3G to aspartic acid rendered the protein insensitive to SIVagm Vif but sensitive to HIV-1 Vif [45]–[48]. Interestingly, the Vif proteins encoded by HIV-2 isolates and by SIVmac239 can target both human and Agm APO3G proteins indicating that their interaction is not restricted by amino acid 128. Assuming that all Vif proteins target APO3G through essentially similar mechanisms, these results suggest that amino acid 128 in APO3G is not the sole determinant for species-specific targeting by Vif but may involve other yet undefined domains in APO3G.

As far as Vif is concerned, we speculated that the species-specific inhibition of APO3G required specific sequences in Vif that mediate the interaction with APO3G. In that regard it is interesting to note that all Vif molecules exhibit regions of homology in their N-terminal and central regions but are quite distinct in their C-terminal domains [58]. It was therefore possible that the determinants for specifies specificity reside in the non-homologous C-termini of the Vif proteins. However, we were unable to verify such a model. In fact, replacing the C-terminal domain of SIVagm Vif by that of SIVmac (AM3) did not result in gain of function against human APO3G (Fig. 3) and, in addition, reduced activity against Agm APO3G (Fig. 4). Conversely, replacing the C-terminal region of SIVmac Vif with that of Agm Vif (AM7) resulted in loss of dual-tropic activity of the SIVmac Vif chimera and retained only partial activity against Agm APO3G. Thus, species-specificity of Vif is not simply determined by the nature of the C-terminal domain.

Our attempts to precisely map the domain(s) in Vif necessary for interaction with APO3G failed since changes in all regions of SIVmac Vif affected its activity towards human APO3G. Interestingly, Vif chimeras that were completely unable to target human APO3G retained significant activity towards Agm APO3G. However, none of the SIVmac/agm chimeras retained as strong an activity against Agm APO3G than the wild type parental Vif isolates. These results suggest that the requirements for Vif to target APO3G are complex and are difficult to explain by a mere adapter function of Vif. Some of the complexity of the Vif/APO3G interaction may arise from the fact that both Vif and APO3G are RNA-binding proteins and that the association of Vif with APO3G could therefore be RNase-sensitive (data not shown). However, the switch from dual- to mono-specific activity of SIVmac/agm chimeras was not caused by a loss of a putative APO3G binding domain in Vif since all the chimeric Vif molecules demonstrated efficient binding to human APO3G in a co-immunoprecipitation analysis (Fig. 3C).

The results of this study lead us to conclude that functional determinants in SIVmac Vif required for targeting human APO3G are distinct from those necessary to target Agm APO3G and are not defined by linear amino acid motifs in Vif. Furthermore, physical interaction of Vif and APO3G may be necessary but is not sufficient for Vif function. Thus, the functional interaction of Vif and APO3G goes beyond a mere physical interaction of the proteins and most likely is a multi-factorial event that may involve additional poorly explored proteins such as CBFβ or yet unidentified cellular factors.
